# Phytochemicals in Garlic Extract Inhibit Therapeutic Enzyme DPP-4 and Induce Skeletal Muscle Cell Proliferation: A Possible Mechanism of Action to Benefit the Treatment of Diabetes Mellitus

**DOI:** 10.3390/biom10020305

**Published:** 2020-02-14

**Authors:** Poonam Kalhotra, Veera C.S.R. Chittepu, Guillermo Osorio-Revilla, Tzayhri Gallardo-Velazquez

**Affiliations:** 1 Departamento de Biofísica, Escuela Nacional de Ciencias Biológicas, Instituto Politécnico Nacional, Prolongación de Carpio y Plan de Ayala S/N, Col. Santo Tomás, CP. Ciudad de Mexico 11340, Mexico; kalhotrapoonam@gmail.com; 2 Departamento de Ingeniería Bioquímica, Escuela Nacional de Ciencias Biológicas, Instituto Politecnico Nacional, Av. Wilfrido Massieu S/N, Col. Unidad Profesional Adolfo López Mateos, Zacatenco, CP. Ciudad de Mexico 07738, Mexicoosorgi@gmail.com (G.O.-R.)

**Keywords:** diabetes mellitus, dipeptidyl peptidase-4 inhibition, garlic extract, ultrasonic-assisted extraction, serine protease inhibition

## Abstract

Diabetes mellitus is a severe health problem in Mexico, and its prevalence is increasing exponentially every year. Recently, DPP-4 (dipeptidyl peptidase-4) inhibitors have become attractive oral anti-hyperglycemic agents to reduce the pathology of diabetes. Gliptin’s family, such as sitagliptin, vildagliptin, and alogliptin, are in clinical use to treat diabetes mellitus but possess side effects. Therefore, there is a specific need to look for new therapeutic scaffolds (biomolecules). Garlic bulb is widely used as a traditional remedy for the treatment of diabetes. The garlic extracts are scientifically proven to control glucose levels in patients with diabetes, despite the unknown mechanism of action. The aim of the study is to investigate the antidiabetic effects of ultrasonication assisted garlic bulb extract. To achieve this, in-vitro assays such as DPP-4 inhibitory and antioxidant activities were investigated. Further, functional group analysis using FTIR and identification of phytochemicals using mass spectrometry analysis was performed. The results showed that 70.9 µg/mL of garlic bulb extract inhibited 50% DPP-4 activity. On top of that, the garlic extract exhibited a 20% scavenging activity, equivalent to 10 µg/mL of ascorbic acid. Molecular docking simulations on identified phytochemicals using mass spectrometry revealed their potential binding at the DPP-4 druggable region, and therefore the possible DPP-4 inhibition mechanism. These results suggest that prepared garlic extract contains phytochemicals that inhibit DPP-4 and have antioxidant activity. Also, the prepared extract induces skeletal muscle cell proliferation that demonstrates the antidiabetic effect and its possible mechanism of action.

## 1. Introduction

Diabetes mellitus (DM) is a condition in people with elevated levels of blood glucose and is a common and major health concern in Mexico [1] and around the world [2]. In clinical use, the majority of antidiabetic drugs typically exist with distinct mechanisms of action but unfortunately possess side effects such as nutritional disorders, weight gain, hypoglycemia, liver damage, and allergic reactions [3]. The elevated incidence of side effects undoubtedly makes investigators and companies look for novel antidiabetic agents with fewer side effects. Recent developments in the pharmacology of diabetes mellitus have led to the discovery of more than one therapeutic drug target to adequately reduce glucose levels and, among these, dipeptidyl peptidase-4 was found to be more effective [4].

**Figure 1:**
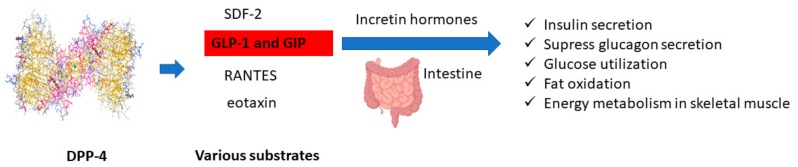
Potential role of protecting incretin hormone degradation by DPP-4 enzyme.

Dipeptidyl peptidase 4 (DPP-4) is a naturally occurring serine protease expressed on the cell surface and thoroughly known to regulate glucose metabolism. DPP-4 rapidly degrades glucagon-like peptide (GLP-1 and GLP-2), glucose-dependent insulin releasing polypeptide GIP [5], stromal-cell derived factor (SDF-2) [6], chemokine (C-C motif) ligand 5 (RANTES) [7], and eotaxin [8]. Among these specific substrates, incretin hormones secreted by intestinal cells are responsible for adequately controlling blood glucose levels [9]. The fundamental purposes of incretin hormones are to naturally stimulate insulin production and inhibit secreted postprandial glucagon, merely delaying gastric emptying and progressively improving pancreatic β-cell function (shown in Figure 1) [10]. DPP-4 inhibitors are scientifically proven to prolong the incretin effect for the successful treatment of diabetes mellitus. 

Currently, GLP-1 receptors have been proven to possess a direct effect on skeletal muscle cells [11] and, upon successful treatment with DPP-4 inhibitors such as vildagliptin, an improvement in energy metabolism was observed [12]. Furthermore, DPP-4 inhibitors of natural origin are proven to stimulate skeletal muscle cell proliferation [13,14]. This promptly led academics and industries to look for the treatment and possible prevention of human diseases by using natural bioactive compounds. There is positively a vast market in the development of functional products due to the growing interest of “healthy,” “pure,” and “clean” foods. Plants and animals serve as a source of enormous, diverse scaffolds [15]. Phytochemicals in plants possess DPP-4 inhibition and are proven to have fewer side effects, are cheaper, and easier to produce at an industrial level [16,17,18,19,20].

In addition, bioactive chemicals from food have proven to be beneficial for improving health because of their functional properties. The bioactive compounds can be naturally antioxidant [21], anti-inflammatory [22], and antifungal [23]. These days, extensive studies on bioactive compounds have promptly led health care sectors and food-based companies to focus collaboratively on the development of functional foods. This functional food contains biomolecules that target specific mechanisms to manage, prevent, and/or treat communicable and non-communicable diseases. Supercritical fluid extraction (SFE) [24], ultrasonic-assisted extraction (UAE) [25], microwave-assisted extraction (MAE) [26], pressurized-liquid extraction (PLE) [27], and enzyme-assisted extraction (EAE) [28] are some of the technologies utilized by academics and industries to extract bioactive molecules from natural sources [29]. 

**Figure 2:**
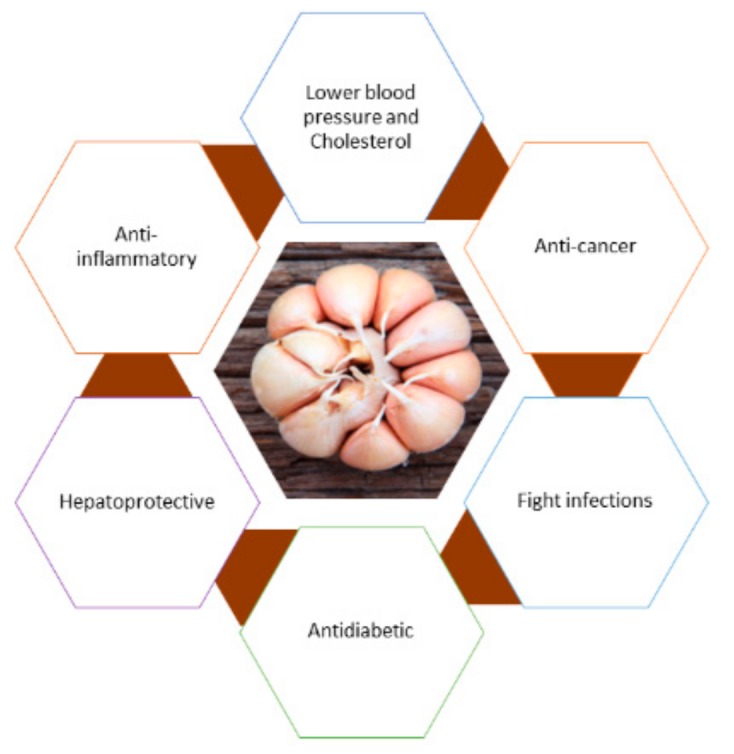
Overall beneficial effects of garlic.

Traditionally, plants are used to manage chronic diseases, and recently extracts in combinations with existing therapeutics are recommended to improve the therapeutic effect. Garlic (*Allium sativum*) naturally belongs to a member of the Alliaceae family, is well recognized as a principal spice, and is additionally used as a remedy for specific ailments and physiological disorders [30]. Figure 2 shows the beneficial effects of garlic proven to lower blood pressure [31], cholesterol [32], combat infections [33], treat metabolic disorders [34], and prevent cancer [35]. Furthermore, garlic extracts are proven to be antidiabetic [36], hepatoprotective [37], anthelmintic [38], anti-inflammatory [39], antioxidant [40], antifungal [41] and wound-healing [42]. The possible mechanism of direct action is still unknown despite the well-known benefits of garlic. The benefits related to diabetes mellitus are reduced blood glucose levels, improved insulin sensitivity, and cardiovascular protection.

In the present study, in vitro anti-diabetic effects of garlic extract were investigated upon dipeptidyl peptidase-4 inhibition and skeletal muscle cell proliferative metabolism. In addition, functional group analysis by Fourier-transform infrared spectroscopy (FTIR), chemical identification using mass spectrometry, and computational studies on identified phytochemicals in garlic extract revealed the possibility to inhibit DPP-4 and its role in the proliferation of skeletal muscle cells.

## 2. Materials and Methods 

### 2.1. Plant Material

Garlic bulbs (*Allium sativum* L.) were purchased from a local market in Mexico City, Mexico. Garlic bulbs were frozen using liquid nitrogen and crushed into a fine powder. The fine powder was immediately used for biomolecule extraction.

### 2.2. Chemicals and Reagents

HPLC grade methanol, dimethyl sulfoxide, 2,2-diphenyl-1-picrylhydrazyl (DPPH), and a human DPP-4 inhibitor screening kit were purchased from Sigma Aldrich (St. Louis, MO, USA).

### 2.3. Preparation of Garlic Extract

A digital ultrasonic bath (G.T.Sonic brand, Model: VGT-1730, Power: 100 W, GuangDong GT Ultrasonic Co., Ltd., Shenzhen, China) was used for the extraction purpose in this study with the frequency of 40 kHz. The extraction was carried out in methanol:water (8:2 *v*/*v*) using a ratio garlic powder:solvent of 1:10 in a 100 mL glass beaker. The beaker was incubated for 30 min in an ultrasonic bath and then filtered using syringe filters of 0.45 µm. The solvent was evaporated under vacuum using an Eppendorf Concentrator 5301 (Eppendorf, Hamburg, Germany), and the resulting residue was dissolved in dimethyl sulfoxide (DMSO) for biological activity assays.

### 2.4. Fourier-Transform Infrared Spectroscopy (FTIR) 

Fourier-transform infrared spectroscopy (FTIR) spectra of garlic extract in aqueous methanol (8:2) and DMSO were recorded at room temperature using a Perkin Elmer Frontier spectrum spectrophotometer, scanning over the frequency range of 4000 to 400 cm^−1^. The spectrum of each sample was acquired using spectrum software version 5.3.1 and a diamond ATR (Perkin Elmer, MA, USA) and each spectrum was an average of 64 scans, with a resolution of 4 cm^−1^. The aqueous methanol aliquots and DMSO aliquots of 10 µL were uniformly spread directly onto the ATR crystal before each spectrum was collected. Functional group analysis was carried out using Know-it-all software (Bio-Rad, Life Science, Hercules, CA, USA).

### 2.5. DPPH Radical Scavenging Activity

The 2,2-diphenyl-1-picrylhydrazyl (DPPH) radical scavenging activity was performed as previously reported [43]. Briefly, 4 mg of DPPH was dissolved in 100 mL methanol. In a 96-well plate, 10 µL plant extract and 190 µL DPPH solution were added to each well and incubated for 30 min at room temperature in the dark. The absorbance at 595 nm wavelength was recorded at room temperature. The degree of scavenging activity was calculated using Equation (1).

(1) $$\%\;scavenging\;activity=\;\frac{\left(\left(Absorbance\;of\;control\right)-\left(Absorbance\;of\;plant\;extract\right)\right)}{\left(Absorbance\;of\;control\right)}\;\times 100$$

### 2.6. Human Dipeptidyl Peptidase-4 (DPP-4) Inhibition Assay

Human dipeptidyl peptidase-4 (DPP-4) inhibition assay was performed in-vitro using a human inhibitor screening kit. The assay was specific to the fluorescent product Gly-Pro that is cleaved upon the binding of DPP-4 and the fluorescence was monitored (λexcitation = 360 nm and λemission = 460 nm) using a Thermo Scientific Varioskan Flash Multimode Reader (Thermo Scientific, Waltham, MA, USA). All the readings were recorded in a dynamic mode for 30 min. Equation (2) was utilized to calculate the relative percent of DPP-4 inhibition, where ΔF/ΔT represents changes in fluorescence over time.

(2) $$\;\;\;\;\;\;\;\;\;\;Relative\;percent\;of\;DPP4\;inhibition=\;\frac{\left(\left(\frac{∆F}{∆T}\right)enzyme-\left(\frac{∆F}{∆T}\right)sample\right)}{\left(\frac{∆F}{∆T}\right)enzyme}\times 100$$

### 2.7. Identification of Phytochemicals in Garlic Extract

The polyphenols in garlic bulb extract were identified using a mass spectrometer (ESI-micrOTOF-Q^TM^, Bruker Daltonics, Bremen, Germany). Negative ion mode was used to acquire the spectra over a mass range of 50–3000 *m*/*z*. The other parameters were capillary voltage 2700 V; dry gas temperature 180 °C; dry gas flow 4.0 L/min; nebulizer pressure 0.4 bar; and spectra rate 1 Hz. Bruker Data Analysis 4.0 software (Bruker Daltonics Inc., Billerica, MA, USA) was used to analyze the data. The native phenolic compounds were assigned based on the matching of molecular weight, isotope pattern, ion mode collected, and considering the polarity of compounds information available in the literature and plant database. 

### 2.8. Activity Atlas Model and Molecular Docking Simulations

In our earlier study [44], the activity atlas model of DPP-4 inhibitors was constructed and validated using Forge software (Cresset Inc., Cambridgeshire, UK). This model was utilized to understand the features of phytochemicals identified in the garlic bulb extract as possible DPP-4 inhibitors.

To perform molecular docking simulations, ensemble docking methodology was utilized in this study using the Flare tool provided by Cresset software (Cresset Inc., Cambridgeshire, UK) [45]. In this study, the DPP-4 in complex with vildagliptin (6B1E), sitagliptin (PDB ID: 4FFW) and anagliptin (PDB ID: 3WQH) were validated initially using structure validation server SAVES and the errors in protein structures were corrected using Flare protein preparation wizard, and the resulting complexes were superimposed. 

The DPP-4 binding site is comprised of a pocket where reference ligands, vildagliptin, sitagliptin and anagliptin, are bound. All the atomics coordinates within the grid box were converged before performing the docking of phytochemicals identified in garlic extract. Discovery studio visualizer 2.5.5 was utilized to understand all the interacting residues and binding pose views between phytochemicals and DPP-4.

### 2.9. Cell Culture, Treatment, and Cell Proliferation Studies

Rat skeletal muscle cells (L6) were cultured as per the procedure described elsewhere [46]. The rat skeletal muscle cells (L6) were cultured in EMEM media, penicillin (100 units/mL), streptomycin (100 µg/mL), and L-glutamine (sterile filtered) at 37 °C in 5% CO_2_ atmosphere. Rat L6 cells with density 3 × 10^3^ cells/well were seeded in a normal 96-well microplate and cultured with 2% horse serum for 2 days until semi confluent was reached, and then the cell was allowed to differentiate for 5 days to form myotubes (80–90% myotubes formed). Resultant myotubes were treated with 5 ng/mL garlic bulb extract for 24 h to determine cell proliferation using sulforhodamine B (SRB) dye assay [47]. After 24 h exposure, 50% aqueous trichloroacetic acid (50 µL) was added to treated cells to fix to plastic substratum, and the resultant plates were incubated for 1 h at temperature 4 °C. Then, the plates were washed with water, air-dried, and then stained with 0.4% SRB dye. The unbound or free SRB dye was removed by washing (with 1% aqueous acetic acid) and bound dye was solubilized by adding 100 µL unbuffered Tris base (10 mM). The absorbance was recorded at 515 nm using Synergy HT Microplate Reader (BioTek Instruments, Winooski, VT, USA). All the experiments were carried out in triplicates.

## 3. Results and Discussion

The use of traditional medicine utilizes a broad range of plants and herbs for the treatment of diabetes mellitus in Mexico and other countries for many years. These plants are considered to play a significantly important role in providing alternative medicine, preventive agents, and as a source of new leads in the process of drug discovery and drug development. Metformin is one of the widely used natural therapeutic drugs to control blood glucose levels in diabetes mellitus, isolated from the *Galega officinalis* plant [48] and, indeed, oral delivery of metformin improves incretin effects by inhibiting the DPP-4 enzyme [49].

**Figure 3:**
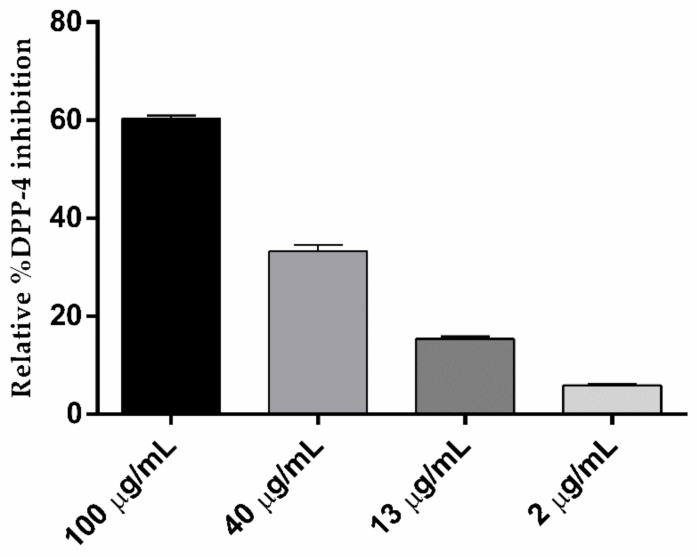
Percent of DPP-4 inhibition of ultrasonic-assisted extraction of garlic in aqueous methanol. Data are expressed as mean ± S.D.

In recent years, DPP-4 has emerged as an important therapeutic drug target to treat diabetes mellitus, and great effort by academics and the pharmaceutical industry have naturally led to the development of DPP-4 inhibitors with few side effects in comparison to current FDA approved therapeutics. During the process of plant screening, garlic was found to be more attractive because of its antidiabetic activity, even though the mechanism of action is unknown. A highly specific fluorometric assay was utilized in this study to determine precisely the DPP-4 inhibition activity by garlic bulb extract. The DPP-4 inhibitory activity of prepared garlic bulb extract in this study is shown in Figure 3 It is observed that the garlic extract showed 60.5% DPP-4 inhibition at 100 µg/mL and the inhibition capacity is concentration-dependent. The results obtained demonstrated a stronger activity in DPP-4 inhibition by prepared garlic bulb extract whose IC50 value is 70.9 µg/mL.

In comparison with methanol extract of green tea (IC50 = 5.3 mg/mL), black tea (IC50 = 6.4 mg/mL) and white tea (IC50 = 0.2 mg/mL), the garlic bulb extract obtained in this work possessed significantly higher DPP-4 inhibitory capacity (IC50 = 70.88 µg/mL). The inhibition of human serine protease dipeptidyl peptidase-4 activity obtained in this study could explain the possible mechanism of the already-observed reduction in blood glucose levels by garlic bulb extract. This inhibition progressively improved the incretin effect and helped in the production of insulin [50].

On the other hand, it is well known that free radicals oxidize many biological components of cells such as proteins, DNA, and lipids, which leads to cell death [51] and finally damages the tissues [52]. Antioxidant activity neutralizes the free radicals and prevents pathogenesis as well as diabetic complications [53,54]. Hence, the garlic bulb extract scavenging ability using the DPPH radical was investigated. It was observed that the prepared garlic bulb extract showed a 20% scavenging activity, which is equivalent to 10 µg/mL ascorbic acid.

**Figure 4:**
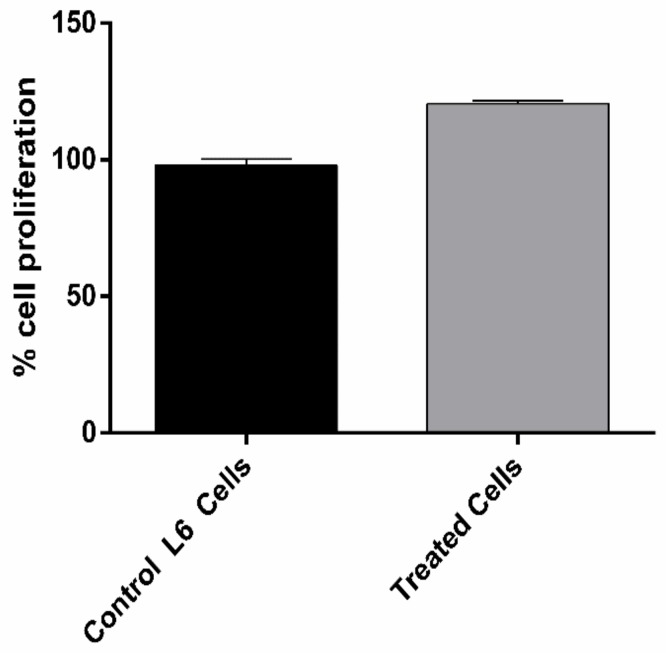
Cell proliferation studies of skeletal muscle cell lines treated with garlic bulb extract (5 ng/mL) and untreated cells for 24 h. All data in the graph are represented as the mean ± S.D of triplicates. Two-tailed unpaired *t*-test was used to calculate significant difference at *p* < 0.05.

Traditional medicinal plants are used to maintain health and serve as an excellent source of new scaffolds for investigators [55,56]. Besides these advantages, plant extracts could exhibit a cytotoxic effect [57]. Thus, the potential cytotoxic studies are necessary to demonstrate the safe use of traditional medicinal plants. To investigate the cytotoxicity of garlic bulb extract in this work, cell proliferation studies were carried out on differentiated skeletal muscle cells. Figure 4 shows the percent of cell proliferation corresponding to differentiated skeletal muscle cells when exposed to 5 ng/mL of prepared garlic bulb extract.

The results In Figure 4 reveal that, upon exposure to prepared garlic bulb extract, there is an increase in cell proliferation by 25% when compared to untreated cells, and this increase behavior is significant (*p* < 0.05). Based on these, it can be concluded that garlic bulb extract is not acutely toxic to cells. Since garlic extract participates in proliferating differentiated skeletal muscle cells, it can be proposed that it altered cellular metabolism by increasing the rate of glycolysis [58]. This is supported by a few investigations that revealed that garlic extract helps in glucose uptake in skeletal muscle cells [59] and reduces blood glucose levels in diabetic animal models [36,60]. On this account, prepared garlic bulb extract provides a therapeutic opportunity to treat diabetes mellitus by proliferating skeletal muscle cells.

**Figure 5:**
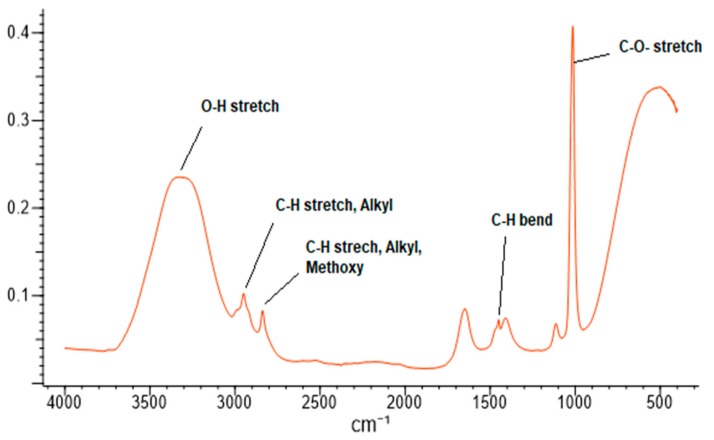
FTIR bands of garlic bulb extract.

To understand the functional groups present in garlic bulb extract, FTIR spectroscopy was carried out. The spectrum of garlic extract is presented in Figure 5. The band between 1800–800 reveals the chemical fingerprint of garlic extract specifically to polyphenols, which is also supported by Xiaonan Lu et al. [61]. The bands between 1125–1000 cm^−1^ (C–O stretch), 1475–1415 cm^−1^ (C–H bend), 2975–2845 cm^−1^ (C–H stretch), 3450–3225 cm^−1^ (O–H stretch) reveal the presence of a hydroxy group (R–OH) in garlic extract. Also, the bands between 1060–1020 cm^−1^ (C–S=O sulfoxy stretch) reveal the presence of aliphatic ether (R–O–R) or sulfoxide (R–(S=O)–R) functional groups in the garlic extract. In addition, the band at 1589 cm^−1^ and 1559 cm^−1^ correspond to the C–C stretch of phenyl and ring base [62].

**Figure 6:**
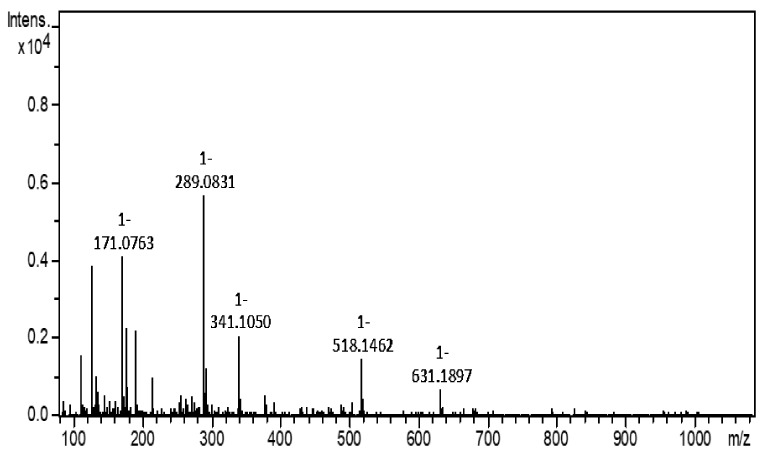
Garlic bulb extract *m*/*z* peaks detected in mass spectrometer (in negative mode).

Based on the functional group analysis in garlic bulb extract, it is clear that hydroxyl and phenolics functional groups contributed to the biological activity DPP-4 inhibition, scavenging activity, and cell proliferative metabolism. Bozin et al. [63] and Rasul et al. [64] also demonstrated the presence of phytochemicals in aqueous methanol extract of garlic. Hence, the mass spectrometer was utilized to identify the phytochemicals in the prepared extract. The resulting extract was directly injected into the mass spectrometer to identify phytochemicals. The qualitative analysis showed the main compounds present in garlic extract detected in negative mode (Figure 6). The following compounds were identified and assigned based on existing phytochemical database: catechin ([M − H]^−1^ *m*/*z* 289.07), caffeic acid 4-O-glucoside ([M − H]^−1^ *m*/*z* 341.10), malonylgenistin ([M − H]^−1^ *m*/*z* 518.11), and calenduloside E ([M − H]^−1^ *m*/*z* 631.3). Since it was already proven that garlic extract inhibits DPP-4, and the main compounds in this extract are the ones mentioned above, this led us to hypothesize that these phytochemicals could probably inhibit DPP-Among these compounds, polyphenol catechin was already proven to inhibit DPP-4 [65].

To understand the potential DPP-4 inhibition of the remaining identified phytochemicals in garlic extract, in silico methodologies such as field-based virtual screening and molecular docking simulations were carried out based on our earlier works [44,66], where the qualitative structure-activity relationship model of DPP-4 inhibitors was constructed and validated experimentally.

The prebuilt QSAR model was utilized to demonstrate the potential DPP-4 inhibition capacity of the identified phytochemicals in garlic bulb extract. Initially, the activity cliff summary had assigned novelty scores to the natural compounds. Results revealed all the chemicals maintained a novelty score of very high, and Table 1 shows the similarity score of the identified phytochemicals predicted by the QSAR model for DPP-4 inhibition.

**Figure 7:**
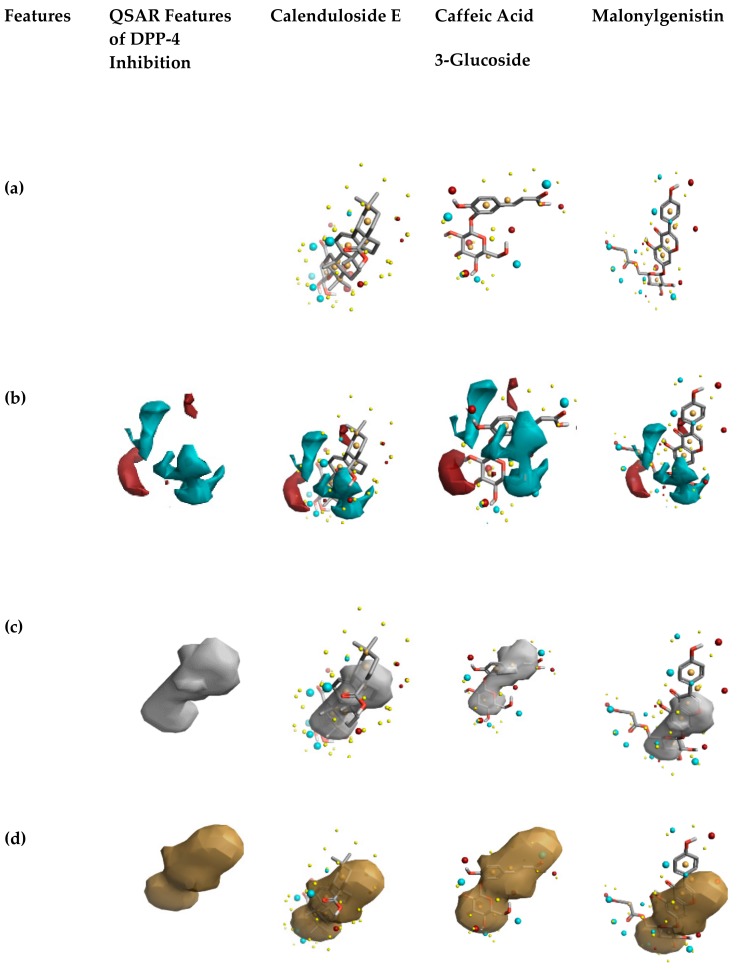
The activity atlas model revealing insights into DPP-4 inhibitory activity. (**a**) 3D structures of phytochemicals identified in garlic bulb extract. (**b**) Activity cliff summary features of DPP-4 inhibitors and phytochemicals of garlic bulb extract. In this, red color shows the positive electrostatic field, and blue color represents the negative electrostatic field responsible for DPP-4 inhibition. (**c**) Average shapes of activity of DPP-4 inhibitors and natural compounds of garlic bulb extract. (**d**) Average hydrophobics of active shape of natural compounds of garlic bulb extract and DPP-4 inhibitors.

The activity cliff summary of positive and negative electrostatic features of the identified phytochemicals such as the DPP-4 inhibitor is shown in Figure 7, compared to already-built QSAR features of DPP-4 inhibition [44,66]. The red color site represents positive electrostatics features, and blue color denotes negative electrostatic features of DPP-4 inhibition. A comparison of the field differences among the three phytochemicals reveals that caffeic acid 3-glucoside possesses increased electronegative and electropositive features. The remaining two phytochemicals possess similar features responsible for DPP-4 inhibition (shown in Figure 7b).

Finally, comparing the features of the hydrophobic shapes of the constructed activity atlas model [44,66] and the identified phytochemicals in the garlic extract, it is observed that they possess increased hydrophobic shape features (shown in Figure 7d). Regarding the features corresponding to average activity shape, Figure 7c shows that the phytochemicals in the garlic extract and QSAR model possess similar shapes. It is clear that the phytochemicals in garlic extract possess features similar to already existing DPP-4 inhibitors. To understand further the phytochemicals interactions with DPP-4, molecular docking simulation was performed.

In this study, ensemble docking was employed, and the docking protocol used was validated using a redocking methodology. It was observed that the Lead Finder docking algorithm [67] could replicate interacting residues to the structures determined using crystallography of vildagliptin, sitagliptin, and anagliptin. The scoring extra precision docking method was used to calculate free energy (ΔG) values of 10 different poses of the remaining three phytochemicals identified in garlic bulb extract. The calculated binding energies are shown in Table 2. 

**Figure 8:**
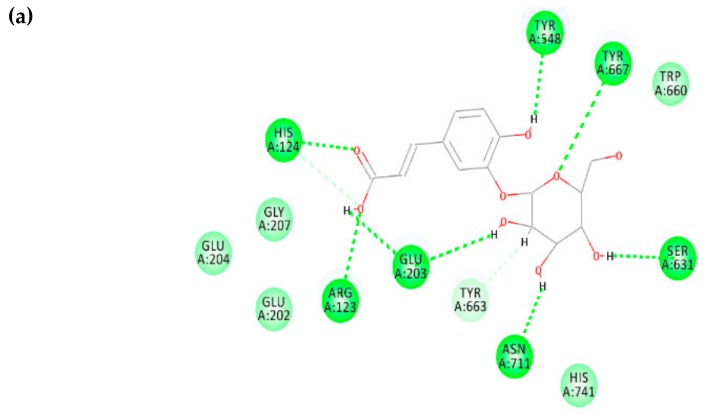

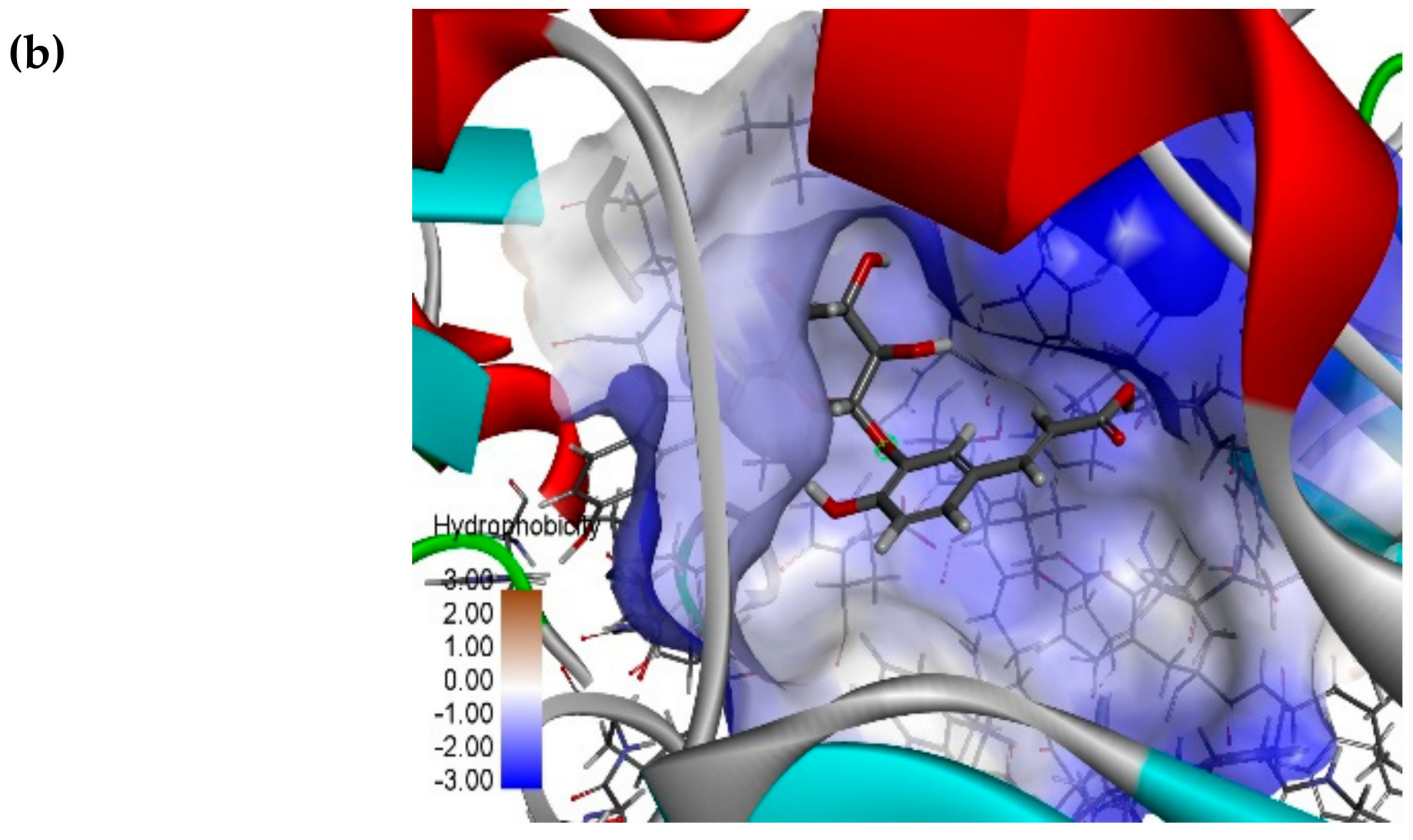
Phytochemical caffeic acid 3-glucoside bound structure on the DPP-4 druggable region with active site residues interacting analysis. (**a**) Two-dimensional interaction analysis, (**b**) binding pose view.

Caffeic acid 3-glucoside possesses the binding free energy of −7.436 kcal/mol and the interacting residues involved at the binding site are TYR 548, TYR 667, TRP 660, SER 631, ASN 711, HIS 741, TYR 663, GLU 203, ARG 123, GLU 202, GLY 207, and GLU 204 (shown in Figure 8). Further non-bonding interaction analysis between caffeic acid 3-glucoside and the DPP-4 active site revealed strong hydrogen-bonding interactions with residues ASN 711, SER 631, GLU 203, TYR 548, GLU 203, TYR 663, ARG 123, HIS 124, and TYR 667. Since caffeic acid 3-glucoside possesses a binding affinity to DPP-4 and the interacting residues belong to the druggable region of DPP-4 (Figure 8), it can be proposed as a new lead to inhibit DPP-4 and treat diabetes mellitus.

**Figure 9:**
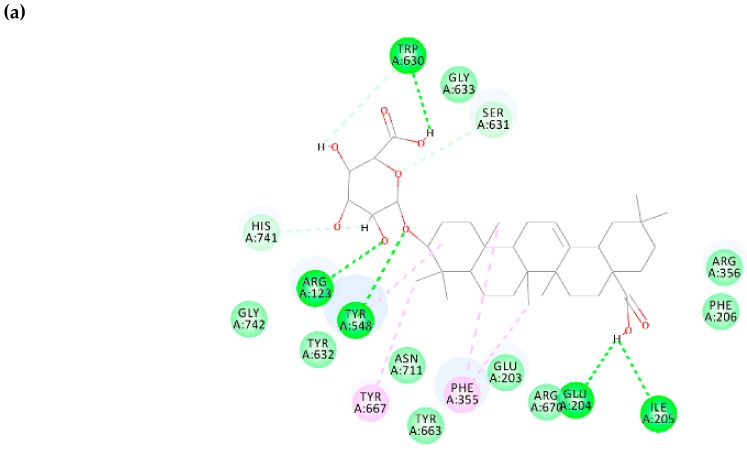

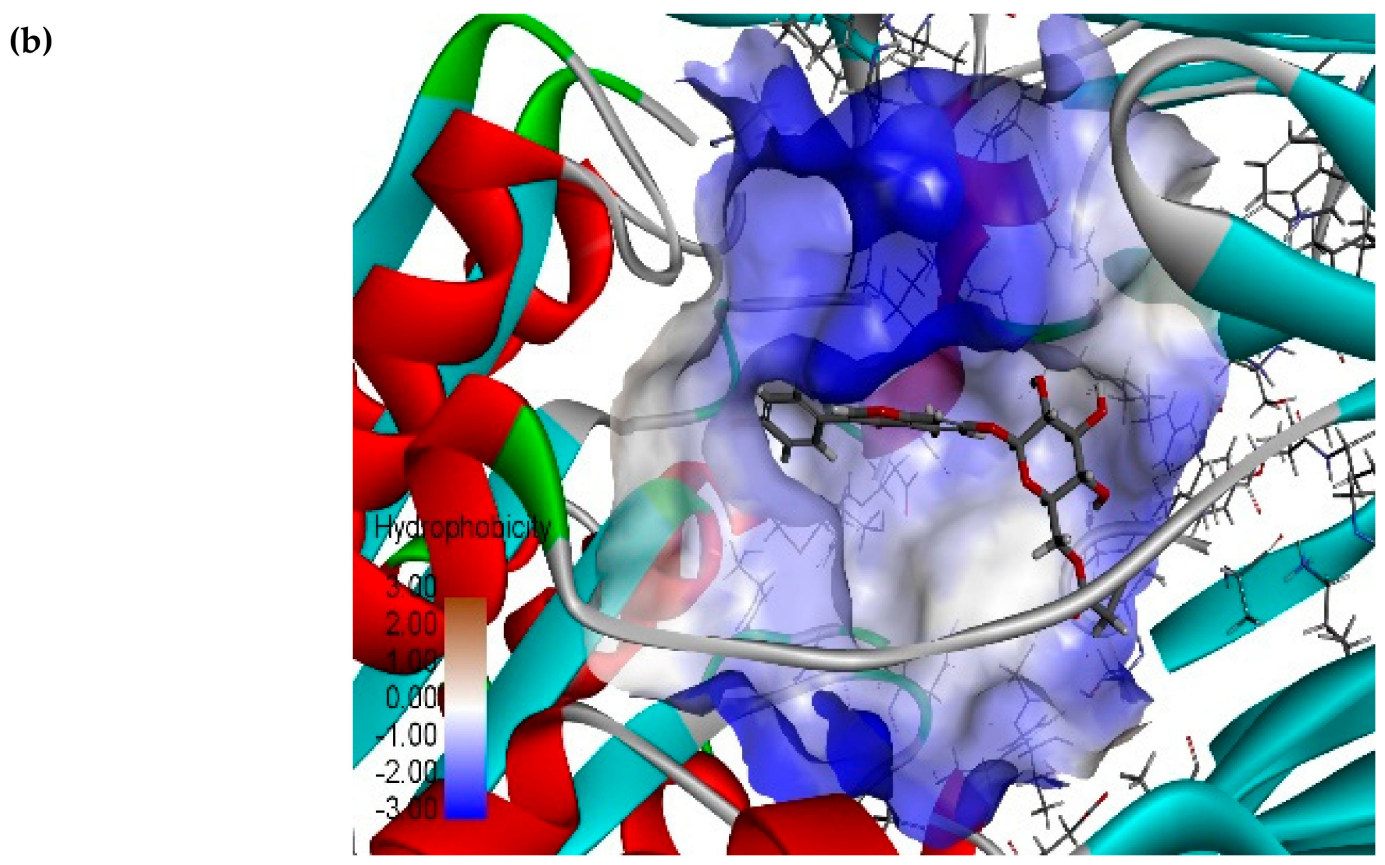
Phytochemical malonylgenistein bound structure on DPP-4 druggable region with active site residue interacting analysis. (**a**) Two-dimensional interaction analysis, (**b**) binding pose view.

Regarding the polyphenol malonylgenistein, it possesses a binding free energy of −7.438 kcal/mol, which is similar to caffeic acid 3-glucoside and the interacting residues involved at the binding site are TRP 630, GLY 633, SER 631, ARG 356, PHE 206, GLU 204, ILE 205, ARG 670, GLU 203, PHE 355, TYR 663, ASN 711, TYR 667, TYR 548, ARG 123, GLY 742, and HIS 741 (shown in Figure 9). Studies on non-bonding interacting analysis of polyphenol malonylgenistein and the DPP-4 interacting site revealed the following: hydrogen bond interactions with residues VAL 207, ARG 358, SER 630, SER 209, PHE 357, and TYR 662; negative electrostatic interactions in specific π-anion with residues GLU 205; hydrophobic interactions with residues TYR 662 and HIS 740 (π-π stacked), TYR 666 (π-π T shaped), and VAL 711 (π-alkyl). Based on the above, it can be proposed that malonylgenistein has a high probability to inhibit DPP-This can be supported by the fact that genistein has been proven to inhibit DPP-4 (IC50 = 0.48 µM), and have antidiabetic properties [68]. Malonylgenistein being a derivative of genistein, it is very probable that it also has the same functional properties.

**Figure 10:**
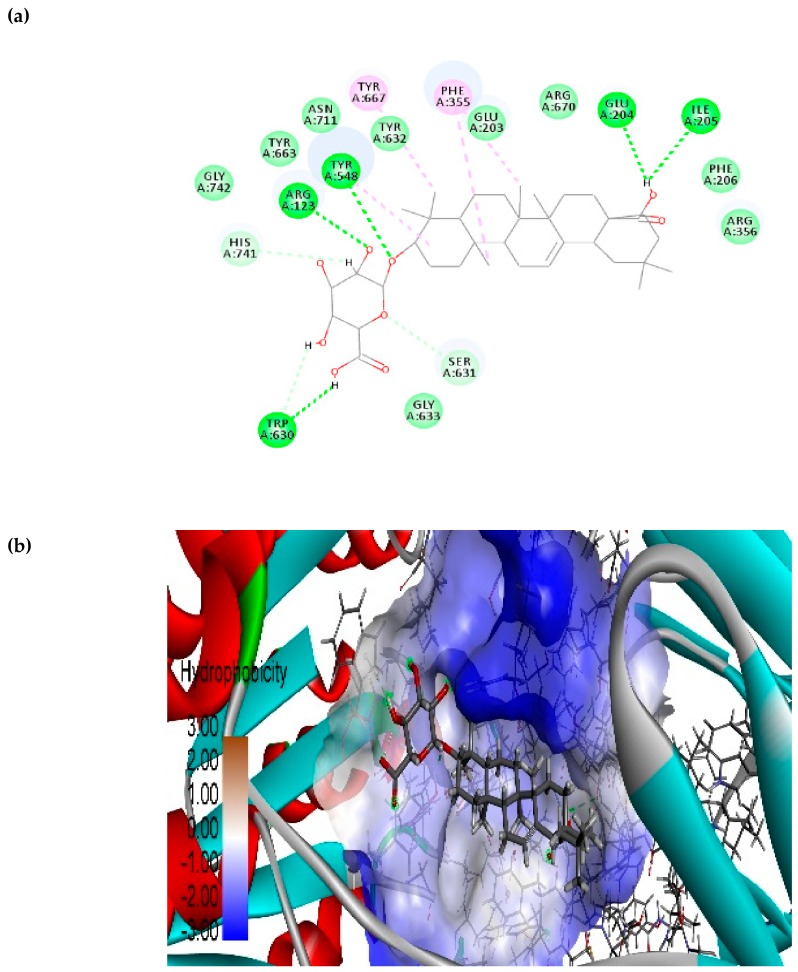
Phytochemical calenduloside E bound structure on DPP-4 druggable region with active site residue interacting analysis. (**a**) Two-dimensional study interaction analysis, (**b**) binding pose view.

Finally, the phytochemical calenduloside E (oleanolic acid 3-*O*-beta-d-glucosiduronic acid) possesses a binding free energy of −10.172 kcal/mol and the interacting residues involved at the binding pocket of DPP-4 are HIS 741, ARG 123, TYR 548, TYR 632, PHE 355, GLU 203, ARG 670, GLU 204, ILE 205, PHE 206, ARG 356, SER 631, GLY 633, TRP 630, and TYR 667 (Figure 10). Besides, a non-bonding interacting residue analysis of the calenduloside E and DPP-4 interacting site revealed the following: stronger hydrogen bond interactions with residues GLU 204, ILE 205, TRP 630, ARG123, and TYR 548 (conventional hydrogen bonding); carbon-hydrogen bonding with interaction residues HIS 747 and SER 631; hydrophobic interactions with PHE 355, TYR 548 and TYR 667 (π-alkyl). Based on the above, it can be proposed that calenduloside E also has a high probability to inhibit DPP-4.

Calenduloside E is a derivative of oleanolic acid, which has been proven to be present in garlic extract [69] and to have antidiabetic properties [70]. The findings in this study show that calenduloside E present in garlic extract probably possesses DPP-4 inhibition activity, which could be the possible mechanism of action to reduce glucose levels in diabetes mellitus.

## 4. Conclusions

Traditional plants are a source of bioactive compounds with diverse scaffolds, well known to treat and manage metabolic disorders like diabetes mellitus. In this study, ultrasonic-assisted garlic extract has been demonstrated as a promising anti-diabetic agent by inhibiting therapeutic drug target dipeptidyl peptidase 4, also having scavenging activity, with the capacity to induce skeletal muscle cell proliferation. FTIR, mass spectrometry and in silico studies reveal the presence of phytochemicals in garlic extract that are probably responsible for DPP-4 inhibition and provide a new mechanism of action of garlic extract to be used for treatment and management of diabetes mellitus. However, further studies related to the inhibition of DPP-4 and hypoglycemic activity of individual compounds present in the prepared garlic extract need to be evaluated for the possible antidiabetic usage.

## Figures and Tables

**Table 1:** Similarity score and novelty score of phytochemicals in garlic extract predicted by activity atlas model for DPP-4 inhibition.

Phytochemical Name	Similarity Score to Field Template	Novelty Score
Caffeic acid 3-glucoside	0.43	Very high
Calenduloside E	0.4	Very high
Malonylgenistin	0.41	Very high

**Table 2:** Calculated binding energies between DPP-4 and phytochemicals of garlic bulb extract.

Phytochemical Name	Protein–Ligand Binding Free Energy Δ*G* (kcal/mol)
Caffeic acid 3-glucoside	−7.436
Calenduloside E	−10.172
Malonylgenistin	−7.438
